# A Robust and Fast/Multiplex Pharmacogenetics Assay to Simultaneously Analyze 17 Clinically Relevant Genetic Polymorphisms in *CYP3A4*, *CYP3A5*, *CYP1A2*, *CYP2C9*, *CYP2C19*, *CYP2D6*, *ABCB1,* and *VKORC1* Genes

**DOI:** 10.3390/ph15050637

**Published:** 2022-05-22

**Authors:** Camille Tron, Régis Bouvet, Marie-Clémence Verdier, Fabien Lamoureux, Benjamin Hennart, Christèle Dubourg, Eric Bellissant, Marie-Dominique Galibert

**Affiliations:** 1 Pharmacology Department, CHU Rennes, Inserm, EHESP, Irset (Institut de Recherche en Santé, Environnement et Travail)-UMR_S 1085, Univ Rennes, F-35000 Rennes, France; marie-clemence.verdier@chu-rennes.fr (M.-C.V.); eric.bellissant@chu-rennes.fr (E.B.); 2 Department of Molecular Genetics and Genomics, Rennes Hospital University, F-35000 Rennes, France; regis.bouvet@chu-rennes.fr (R.B.); christele.dubourg@chu-rennes.fr (C.D.); marie-dominique.galibert-anne@univ-rennes1.fr (M.-D.G.); 3 Laboratory of Pharmacology, CHU Rouen, F-76000 Rouen, France; fabien.lamoureux@chu-rouen.fr; 4 CHU Lille, Service de Toxicologie et Génopathies, F-59000 Lille, France; benjamin.hennart@chru-lille.fr; 5 CNRS, IGDR (Institut de Génétique et Développement de Rennes)-UMR 6290, Univ Rennes, F-35000 Rennes, France

**Keywords:** pharmacogenetics, panel, multiplex, CYP450, personalized medicine

## Abstract

In the field of pharmacogenetics, the trend is to analyze a panel of several actionable genetic polymorphisms. It may require the use of high-throughput sequencing which demands expensive reagents/instruments and specific skills to interpret results. As an alternative, the aim of this work was to validate an easy, fast, and inexpensive multiplex pharmacogenetics assay to simultaneously genotype a panel of 17 clinically actionable variants involved in drug pharmacokinetics/pharmacodynamics. We designed primers to perform a multiplex PCR assay using a single mix. Primers were labeled by two fluorescent dye markers to discriminate alleles, while the size of the PCR fragments analyzed by electrophoresis allowed identifying amplicon. Polymorphisms of interest were CYP3A4*22, CYP3A5*3, CYP1A2*1F, CYP2C9*2-*3, CYP2C19*2-*3-*17, *VKORC1*-1639G > A, *ABCB1* rs1045642-rs1128503-rs2229109-rs2032582, and CYP2D6*3-*4-*6-*9. The assay was repeatable and a minimum quantity of 10 ng of DNA/ sample was needed to obtain accurate results. The method was applied to a validation cohort of 121 samples and genotyping results were consistent with those obtained with reference methods. The assay was fast and cost-effective with results being available within one working-day. This robust assay can easily be implemented in laboratories as an alternative to cumbersome simplex assays or expensive multiplex approaches. Together it should widespread access to pharmacogenetics in clinical routine practice.

## 1. Introduction

Pharmacogenetics aims at improving drug response by looking for genetic polymorphisms that are involved in drugs pharmacokinetics or pharmacodynamics. Several pharmacogenes have been associated with efficacy or toxicity of many medicines such as immunosuppressant, antipsychotics, antidepressant, anti-infectious, anticoagulants, or analgesics [1,2,3,4,5]. For some of these medicines, dosage adjustment guidelines have been reported by learned societies to individualize treatment according to the patient genotype [6]. Pharmacogenetics can also be useful in the context of forensic toxicology to elucidate overdose cases. It may also explain variability in the intensity of side effects that occur in case of misuse of drugs that act on the central nervous system [7,8]. Genes of interest are mostly those coding for cytochromes P450 (CYP450) and some membrane transporters since these proteins are strongly involved in many drug metabolic pathways [9].

Clinical implementation of pharmacogenetics is a valuable approach in the era of personalized medicine to optimize drug safety and efficacy. The list of clinically relevant pharmacogenetics biomarkers (mostly single nucleotide polymorphisms (SNP)) is increasing, and patients are likely to be treated by drugs from different therapeutic classes along their medical history. It is thus of utmost importance for laboratories to gain in efficiency and be able to simultaneously genotype a set of actionable pharmacogenes instead of performing cumbersome individual experiments per gene [10]. Next generation sequencing (NGS) is a high throughput sequencing technology that offers a very robust solution to determine in parallel specific genomic variation. Targeted NGS is thus expected to be the technology of choice to sequence and genotype tens of genomic variations of interest including drug metabolizing enzymes [11,12]. This technology has revolutionized our practice and is very well adapted in the context of pharmacogenomics studies where numerous genes and patient samples have to be analyzed or for high throughput platforms that gather many samples in a routine practice. However, data processing and analysis in a high throughput setting requires specific bioinformatics skills to be acquired by laboratories. Reagents and instruments may be expensive for some labs below a minimum threshold of samples to analyze, which suggests that other approaches may be more adapted to low/mid throughput screening [12]. Technologies based on fluorescent dyes and real-time PCR are fast and convenient to analyze a limited number of genetic hotspots [13]. Yet, a single reactional mix must be prepared for each SNP increasing the cost, the turn-around time, and the risk of errors when several polymorphisms ought to be analyzed for the same patient. In order to overcome these hurdles and as an alternative to the above-mentioned assays, the objective of this study was to develop a robust and fast multiplex genotyping assay suitable to identify a panel of genetic variants of utmost interest in pharmacology and toxicology.

## 2. Results

We defined a panel of 17 genetic polymorphisms selected for their association with drug pharmacokinetics, drug response or when dosing recommendations were available in international databases [6,14,15,16,17,18,19,20,21,22,23]. The assay was designed to identify clinically relevant variants in genes coding for drug metabolizing enzymes from the CYP450 system and for the membrane transporter ATP-binding cassette 1 (ABCB1 or P-gp) since many drugs are substrate of this efflux pump. It included bi-allelic and tri-allelic SNP and nucleotide deletions. Table 1 reports the selected variants and gives an overview of the molecular consequences and some examples of drugs impacted by these genetic polymorphisms.

### 2.1. Optimization of the Assay

**Figure 1:**
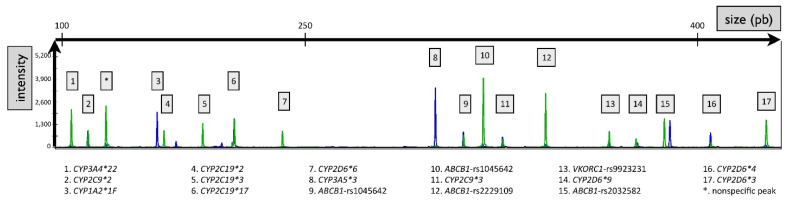
Resolution of the 17 amplicons corresponding to the clinically relevant genetic polymorphisms. Example of an electrophoregram obtained for one DNA sample. The green peaks correspond to the fluorescence of the HEX probe. The blue peaks correspond to the fluorescence of the FAM probe.

As mentioned previously, the pharmacogenetic panel includes several members of the CYP450 family that share sequence similarity. Therefore, PCR primers were carefully designed to be specific to each position of interest leading to distinct size amplification ranging from 100 bp to 500 bp amplicon. It is noteworthy that *CYP2D6* has two pseudogenes *CYP2D7* and *CYP2D8*, which share up to 94.2% and 89.1% sequence similarity with *CYP2D6*, respectively. To accurately genotype *CYP2D6*, forwards-PCR primer targets a specific sequence of *CYP2D6* absent in *CYP2D7* and *CYP2D8* sequences. In addition, to discriminate Wild-Type (WT) and variant allele, forward primers differed respectively from their terminal nucleotide (3′ end). As described in our previous work a nucleotide substitution was inserted at position −4 or −3 of each forward primer-3′ end, in order to destabilize the binding and major WT and Variant primer binding specificity promoted by the terminal nucleotide [24]. Finally, the concentrations of primers were optimized to reach comparable signals between WT and variant genotypes (ratio between intensities of the dyes FAM/HEX ≈ 1 for all polymorphism positions) and to obtain signals at least 10 times higher than the background noise (raw intensity >100). This led to the generation of a dedicated 17 variants-panel, screening for 14 single nucleotide substitutions, and 3 deletions of nucleotides. As shown in Figure 1, the electrophoregram resolution allowed to discriminate each amplicon and showed quantifiable signal intensity.

**Figure 2:**
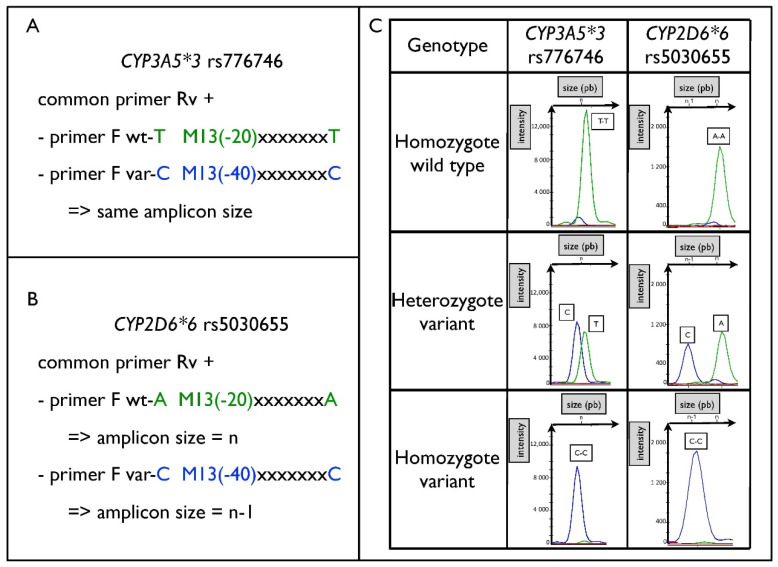
Resolution of a bi-allelic polymorphisms. Primer design for *CYP3A5* rs776746 (**A**). Primer design for *CYP2D6* rs5030655 (**B**). Representative electropherogram results of the migration of fragments according to genotype (**C**). *CYP3A5* rs776746 illustrates results of bi-allelic discrimination for a single-nucleotide polymorphism. *CYP2D6* rs5030655 illustrates results for a polymorphism based on the deletion of 1 nucleotide. The green peak corresponds to the fluorescence of the HEX probe (WT) and the blue peak corresponds to the fluorescence of the FAM probe (Variant).

**Figure 3:**
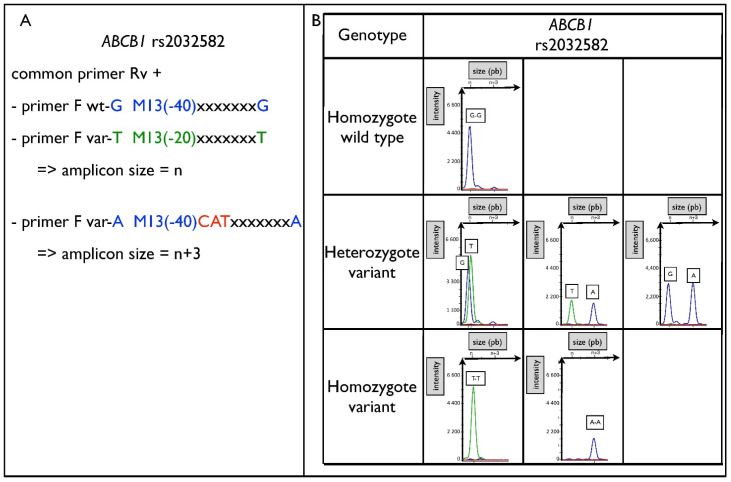
Resolution of tri-allelic SNP. Primer design for *ABCB1* rs2032582 (**A**). Representative electrophoregram results of the migration of fragments according to genotype (**B**). *ABCB1* rs2032582 illustrates results of tri-allelic discrimination for a single-nucleotide polymorphism. The green peak corresponds to the fluorescence of the HEX probe and the blue peak corresponds to the fluorescence of the FAM probe.

Within the 14 single nucleotide substitutions, 13 are bi-allelic, while the SNP, *ABCB1*-rs2032582 is tri-allelic. The 13 bi-allelic SNP can easily be identified using two specific forward primers labeled with two distinct fluorescent dyes (FAM, Hex fluorescence) and a common reverse primer (Figure S1). As expected, the fragment analysis of the three deletions of nucleotides lead to a different migration size between wild type vs. variant amplicon. The electrophoregrams of each genotype for a bi-allelic SNP (e.g., *CYP3A5* rs776746) or a deletion (e.g., CYP2D6*6 rs5030655) are shown in Figure 2. The good resolution of the capillary electrophoresis is used to discriminate the tri-allelic SNP (*ABCB1*-rs2032582 G or A or T) by generating two different size amplicons (n and n + 3) using as above a common reverse primer and three distinct forward primers (labeled with FAM or HEX) as follows: *ABCB1*-rs2032582-T (HEX fluorescence), *ABCB1*-rs2032582-G and *ABCB1*-rs2032582-A (FAM fluorescence). The *ABCB1*-rs2032582-T (HEX fluorescence) and the *ABCB1*-rs2032582-G (FAM) generate an identical amplicon length of n nucleotides (n), while the HEX-*ABCB1*-rs2032582-A forward primers generates an amplicon length increase of three nucleotides (n + 3). The electrophoregrams of each *ABCB1*-rs2032582 G or A or T genotype are illustrated in Figure 3.

### 2.2. Validation of the Assay

Repeated analysis using a set of eight internal DNA controls (50 ng) gave the same genotype results on intraday experiments (duplicate) and inter-day experiments (4 days) Table S1. Next, we determined the range of DNA required for accurate screening according to the signal intensity (a threshold of 100 being defined as acceptance criterion). As anticipated, we found that the amount of DNA modified the performance of the analysis. As shown in Table 2, peak intensities were all >100 with a minimal amount of 10 ng of DNA. Signal intensity obtained for 250 ng and 500 ng of DNA were equivalent presumably due to excess DNA compared to PCR reagents. Together this shows that the genotypes can be accurately determined when DNA sample amounts range from 10 to 250 ng. Outside this range, the intensity of the signal does not meet the acceptance criteria to interpret migration fragment data. Moreover, no inter-sample contamination was observed since no signal was detected at the position of the blank samples inserted between the DNA samples (data not shown).

The stability of the ready-to-use pool of primers and fluorescent probes at the working conditions was validated for five freeze–thaw cycles. Indeed, genotyping results were similar in an experiment performed with a freshly prepared pool and in another experiment performed with an aliquot frozen and thawed five times. Although the peak intensities of fragments were lower when a freeze–thawed reagent was used, they remained >100 intensity units (Table 3). Thus, a pool of primers can be stored at −20 °C and used several times, which is cost effective, convenient, and timesaving.

### 2.3. Genotyping Method Comparison

#### 2.3.1. Evaluation of the Accuracy of the Assay Compared to Reference Methods

A retrospective cohort of 121 DNA samples, was screened using the 17 variants-PCR multiplex panel (hereafter called the “Pharm-17-panel”). This step aimed to assess whether the 17-multiplex genotyping matched the results obtained with reference methods. All genotypes were confirmed without false positive or false negative. Consistency of genotyping results per kind of genotype is reported in Table 4. For each gene, samples carriers of variants and wild type genotype were included. In some cases, the allele was not frequent enough to include homozygous variants samples (i.e., rs35599367). The largest comparison was performed for *CYP3A5* (121 samples) that is one of the most frequently analyzed in clinical practice.

#### 2.3.2. Comparison of Cost and Analytical Workflow with Reference Methods

Regarding the cost of the analysis, we included the cost of labware and reagents. Cost of instrument and personal was not included since it may depend on each center organization. As reported in Table 5, the PCR-multiplex assay is cheaper than the two other methods, even for a large set of samples (48 samples). The workload to perform the PCR multiplex assay leads to a fast turnaround time with results available within 6.5h whereas the analytical parameters remain good enough for the purpose of hotspot analysis.

### 2.4. Clinical Cases

In order to emphasize how patients would benefit from specific and fast track pharmacogenetics implementation, we reported representative patient cases showing the benefit of the new multiplex genotyping method compared to usual workflow.

Having established the accuracy of the Pharm-17-panel to screen for pharmacogenetics markers, we evaluated in routine practice the benefit of such assay. To this end, we reported three representative clinical cases we had in charge in our department using standard protocol.

The first case concerns a patient treated with clozapine and haloperidol to combat schizophrenia. Pharmacogenetic analyses were requested because the patient appeared to be a poor responder to clozapine. Moreover, therapeutic drug monitoring of the antipsychotic showed that plasma concentrations were at the bottom of the therapeutic range despite high dosages (0.2 mg/L at the dosage of 425 mg/day) and could not be explained by drug–drug interaction or non-adherence to treatment. According to clozapine and haloperidol pharmacokinetics [25,26], we explored at that time six relevant pharmacogenes: *ABCB1*, *CYP3A4*, *CYP3A5*, *CYP1A2*, *CYP2D6, and CYP2C19*. Three individual assays were performed in our lab to genotype *ABCB1*, *CYP3A4*, and *CYP3A5* (2 Taqman^®^ and 1 PCR-RFLP assays). The analysis of *CYP1A2*, *CYP2D6, and CYP2C19* were subcontracted and DNA-samples sent out. Results obtained from the different laboratories reported two genetic polymorphisms that may explain clozapine underexposure and resistance. Indeed, the patient was *CYP1A2* *1F/*1F and *CYP2C19* *1/*17. It means that CYP1A2 is highly inducible by external factors such as smoking or caffeine and that she is a rapid metabolizer for CYP2C19 [18,23]. Nevertheless, a limit of this interpretation is the lack of information regarding the smoking status or the caffeine consumption of the patient. The patient was also carrier of one allele of low activity for ABCB1 but the clinical relevance of this variant alone on clozapine pharmacokinetics is unknown. Moreover, the patient was an “intermediate metabolizer” for CYP2D6 which may potentially cause increased plasma concentrations of haloperidol but was not clinically relevant to require dosage adjustment according to the guidelines [19]. The final report of these results obtained from different molecular technologies and different labs was available for the clinician 4 months after the clinical request. The dosage of clozapine was increased up to 650 mg/day and plasma concentrations increased to be within the therapeutic range (0.4 mg/L).

Leftover DNA sample from this patient was re-analyzed using our in-house 17-panel multiplex PCR assay. Results obtained and validated in a very fast time (2 days) were fully concordant with conventional methods (Table S1). With this robust method and its rapid turn-around time, management of the patient treatment would have been possible in a working week.

The second clinical example reports the cases of two pediatric patients, treated with vincristine to combat acute lymphoid leukemia. Both experienced severe neurologic toxicity to vincristine. To explore pharmacokinetic reasons for the intensity of these adverse events, clinicians asked for pharmacogenetics exploration. According to vincristine pharmacokinetic pathway [27], we researched variants in *CYP3A4*, *CYP3A5* and *ABCB1* using three different assays. The first patient was heterozygous carrier of three SNP of *ABCB1* leading to lower activity of the protein, he was carrier of an inactive allele of *CYP3A4* (*22), and was a *CYP3A5* non-expresser. It was hypothesized that this genotype could have been a risk factor of neurotoxicity contributing to decrease vincristine metabolism by CYP3A and to increase drug distribution to the central nervous system (because of lower efflux activity of ABCB1 at the brain barrier [27]). The second patient was homozygous for three variants of *ABCB1*, but was *CYP3A4* wild type and *CYP3A5* non-expresser. Similarly, the lower activity of the membrane transporter ABCB1 could have led to high drug concentrations in the CNS. For both patients, results were expected quickly by clinicians since they wanted to take it into account to choose the dosage of the next chemotherapy cycle of these patients. Reports of these pharmacogenetics tests performed in house were available within 3 weeks.

As previously, we re-analyzed leftover DNA samples and found fully concordant genotypes for the genes of interest (Table S2). With the in-house multiplex assay, all pharmacogenetic analysis carried out for these two patients were performed in a single experiment, with a much faster turnaround time to provide results (a few days). In addition, in one of the pediatric patients, a full deletion of *CYP2D6* was observed by the lack of any peak at the position of *CYP2D6* fragments on the electrophoregram (it was further confirmed by NGS and gel PCR assays). The physician was informed of this incidental analysis in order to tailor in the future the dosage of drugs metabolized by the CYP2D6. Altogether, this supports the added value of such multiplex screening for drug management in a routine practice.

## 3. Discussion

A multiplex PCR assay was successfully developed and validated to genotype simultaneously a panel of 17 genetic polymorphisms known to modify drug responses or pharmacokinetics. To the best of our knowledge, it is the first report of such a genotyping method applied to pharmacogenetics of *CYP450*, *ABCB1,* and *VKORC1*.

We showed that the assay is robust and accurate since a minimal amount of 10 ng of DNA was sufficient to perform the analysis and the consistency of the results with reference methods was complete. Compared to our previous work [20], we were able to optimize the design of the assay to include 17 variants, which is two-times more than our first panel. Moreover, we were able to discriminate a tri-allelic variant with only two fluorescent dyes (rs2032582) and distinct but very close fragments lengths (difference of only three base pairs), demonstrating that the resolution of the capillary electrophoresis was good enough.

The choice of the genes and variants included in the 17-variants panel was based on literature review of the most actionable pharmacogenes recommended by learning society according to their frequency in Caucasian population and their consequences on protein activity. Indeed, many drugs are substrates of CYP450 or ABCB1, and genetic polymorphisms on these proteins may explain a substantial part of pharmacokinetics variability [19]. We added a variant of *VKORC1* since its genetic polymorphisms are important for the management of anticoagulant drugs such as warfarin. *VKORC1* polymorphisms have been shown to account for approximately 25% of the inter-patient variability of warfarin dose requirement [15]. However, we must be aware that the Pharm-17-panel misses some relevant variants that can be more frequent in subjects with non-European ancestry. Moreover, validation of our assay for homozygous carriers of some variants in *CYP2C19*, *CYP2D6*, *CYP3A4,* and *ABCB1*, remained to be evidenced since none of the DNA samples from our cohort were carriers of these rare genotypes.

Combined with our previously described panel more focused on variants relevant for anticancer drugs (including *TPMT*, *NUDT15*, *DPYD,* and *UGT1A1*) [24], the assay reported herein could be valuable to implement preemptive pharmacogenetics testing in clinical practice such as approaches of pharmacogenetics “passport” already used in some countries [10,28,29,30]. Some actionable pharmacogenes such as *CYP2B6* and *SLCO1B1* associated with efavirenz or statins response or toxicity respectively, are not included in the current version of our panel. The reason is the availability of other biomarkers to monitor these treatments in our center such as efavirenz therapeutic drug monitoring and creatine-phosphokinase monitoring that make clinicians not very keen on using pharmacogenetics assays for these drugs. However, there is room to improve the panel by adding these pharmacogenes in the future.

Compared to high throughput NGS screening, a limit of the Pharm-17-panel is that it is not set to analyze rare or exploratory variants [31]. This single assay permits to routinely analyze almost twenty genetic polymorphisms, whereas before we needed more analytical time to perform only two (*CYP3A5* and *CYP3A4*) genotyping with simplex medium throughput methods (e.g., Taqman^®^). Therefore, the Pharm-17-panel appears to be a good compromise to widespread access to pharmacogenetics in medical care.

One weakness of the assay is to be poorly quantitative. Copy number variations (CNV) of *CYP2D6* could not be accurately evidenced. We could suspect homozygote full gene deletion in case of lack of peak on the electrophoregram at the *CYP2D6* position as envisioned for one of the pediatric patient cases reportedpediatric patient-reported cases. *CYP2D6* pharmacogenetics must be interpreted with CNV, since CNV can be responsible for poor or ultra-rapid metabolizer phenotype [32]. To overcome this issue, in case of *CYP2D6* genotyping request, the Pharm-17-panel assay must be completed with another method to detect deletion or duplications of the gene. In our center, we chose a non-quantitative assay to look at duplication or deletion of *CYP2D6* with a method based on PCR and agarose gel migration adapted from Dorado et al. and Puapraset et al. [33,34]. Moreover, consensus recommendations from Pratt et al. on clinical *CYP2D6* Genotyping allele selection defined a minimal set of alleles that should be included in *CYP2D6* pharmacogenetic assay (Tier 1) [32]. All variants corresponding to these alleles are not included in our assay. It means that to accurately genotype this gene we have to use additional methods or to complete our panel (for instance with *10 and *41). We are currently working on this perspective of improvement. Anyhow, our panel remains useful, convenient, and cost effective to look simultaneously in one single experiment at four major star alleles of *CYP2D6*, compared to simplex PCR assays.

Despite the limitations mentioned above, one of the main strengths of the PCR-multiplex assay is its ability to be easily implemented in a lab. It requires universal and affordable reagents and instruments. Importantly, it speeds up the turnaround time allowing to obtain results rapidly (within a working day in case of urgent request). It is therefore of high interest for some clinical issues when genotyping is required for treatment initiation to determine the starting dose or in case of toxicity to tailor the dosage. Moreover, targeted based-panel centralized the genotyping of several relevant genes for a given clinical situation in the same experimental run and in the same lab. There is less need to subcontract analysis and send-out DNA-samples avoiding many pitfalls. First, as illustrated with the clinical cases, the turn-around time with standard analysis and workflow was elevated, sometimes taking several months to obtain all genotyping results. Complete results, explaining inappropriate drug response, were sent with much delay, postponing clinical management. Second, when all analysis cannot be performed in the same experiment or in the same lab, DNA samples are split, which can be challenging in case of a small amount of available sample (i.e., in pediatric patients a small volume of blood is collected). In addition, the probability of analytical error is increased with the number of independent analyses. Third, it is not rare for drugs to be the substrate of several metabolizing enzymes or membrane transporters. The interpretation of pharmacogenetic results requires a global vision of all the analysis to be as relevant as possible. Subcontracting does not ease comprehensive interpretation. Finally, when all genotypings are performed within the same multiplex analytical run, raw data corresponding to each gene can be interpreted in the light of the other ones. It may be worth to refine the interpretation in case of results at the limit of the acceptance criteria of the assay and to avoid some inconclusive results.

Overall, the multiplex assay allows a much more efficient and robust process with a fast feed-back to clinician. Patients will thus benefit earlier from therapeutic optimization. This Pharm-17-panel assay should promote the implementation of pharmacogenetics into clinical practice which is a major challenge of personalized medicine [20,35].

## 4. Materials and Methods

### 4.1. Material and Reagent

Samples used in this study were anonymized DNA leftover samples previously analyzed with a genotyping assay such as Taqman^®^, NGS or PCR-RFLP in certified laboratories from University Hospital centers (Rennes, Lille and Rouen university hospitals). Samples and control DNA were extracted from blood collected either on EDTA or heparinized tubes in humans who gave written consent for use of their samples for research purposes. The study was conducted in accordance with the Declaration of Helsinki, and the protocol was approved by the Ethics Committee of Rennes university hospital (Authorization n 21.112). DNA was extracted from blood with a Microlab STAR liquid Handling System (Hamilton, Courtaboeuf, France) using a Macherey-Nagel^®^ Nucleospin Blood L kit (Hoerdt, France). DNA concentration was measured using a NanoDrop One spectrophotometer (NanoDrop Technologies Inc., Wilmington, DE, USA). The multiplex PCR kit was provided by Qiagen^®^ (Courtaboeuf, France). Primers and fluorescent dyes were provided by Eurofins MWG (Operon, Les Ulis, France). The denaturant Formamide and the dye size internal standard GeneScan™ 500 ROX™ were purchased from Applied BiosystemsTM/Thermo Fisher Scientific (Illkirch, France). PCR reaction was performed on a thermocycler Veriti™ 96-Well Thermal Cycler and samples were analyzed on a sequencer ABI 3500 Genetic Analyzer, both from Applied BiosystemsTM/Thermo Fisher Scientific (Illkirch, France). Data were processed using the GeneMapper^®^ 6.0 software (Applied Biosystems™, Illkirch, France).

### 4.2. Principle of the Assay and Optimization

The assay was adapted from our previous work focused on a panel of pharmacogenes used to prevent anticancer drugs toxicities [24] and derived from a method reported by Schuelke et al. [36]. It consists in a multiplex allele-specific PCR. The first step aimed to optimize the primers sequences to avoid overlap in amplified regions. Sizes of fragments were designed to be specific to each genome region including polymorphisms of interest. Forward primers were specific to the genotype (variant or wild-type) and Reverse primers were the same for both alleles. Primers were associated either with a M13 (−20) or a M13 (−40) universal sequence. Then, fragments were labeled by two different fluorescent dyes (HEX specific to M13 (−20) and FAM specific to M13 (−40)). These two dyes allow discriminating variants or wild type genotypes. The final protocol was as follow: in the same well, 50 ng of DNA sample were mixed with 7 µL of water and 10µL of “Qiagen multiplex PCR mix”, 2 µL of the two dyes (both at 0.7 pmol/μL in the final mix) and primers all pooled in the same solution. Sequences of primers and their respective concentrations in the mix are reported in Table S3. Each primer concentration was optimized to obtain the best signal intensity. Fragments were amplified by PCR including an activation cycle of 95 °C for 15 min, and 33 cycles of amplifications (with the following sequence: 94 °C for 30 s, 58 °C for 90 s, and 72 °C for 60 s). The last cycle was set at 72 °C for 30 min. Then, PCR products (1 µL) were denatured with formamide (18µL) and GeneScan™ 500 ROX™ (0.2 µL) was added in each well. Samples were loaded on the analyzer. Each electrophoregram was visually checked for peak intensity and resolution. We defined the acceptance criterion of the results after migration on the sequencer as follows: the height of the signal of each fragment should be higher than 100 (intensity unit) and lower than 20,000 (intensity unit) to avoid saturation, and the length of each fragment should not differ from +/− 1 base pair from the theoretical length of the fragment. Raw data were automatically processed using GeneMapper^®^ software (version 6) and were interpreted with a local Microsoft Excel^®^ interface providing a user-friendly and synthetic report of genotypes for each gene and samples. Illustration of the principle of the assay is reported in Figure S1.

### 4.3. Validation of the Assay

Before implementation in clinical routine, performances of the assay were assessed to validate its reliability. First repeatability was assessed by genotyping eight internal controls samples of known genotypes in duplicate four times in four independent experiments. This set of eight samples of known genotypes were further used as internal reference control in the other experiments performed to validate the assay. Robustness was checked by testing the influence of the amount of DNA analyzed (from 1 to 500 ng) on the specificity and sensitivity of the results. Inter-samples contamination was evaluated by inserting blank samples in wells (water without DNA) between DNA samples (n = 8 replicates). The stability of the pool solution of diluted primers and fluorescent dyes was checked for five freeze–thaw cycles (−20 °C/ambient temperature). Genotyping results from control DNA samples were compared between samples analyzed with a freshly prepared pool solution and the same samples analyzed with the same pool frozen and thawed five times.

### 4.4. Genotyping Methods Comparisons

#### 4.4.1. Evaluation of the Accuracy of the Assay Compared to Reference Methods

The accuracy of the assay was assessed by comparing genotyping results provided by the multiplex assay compared to those previously reported by reference methods used in our center or in our partner subcontracting centers. The comparison was performed on at least 25 and up to 121 DNA samples per polymorphism included in the panel. Reference methods were Taqman^TM^ assays (Thermo Fisher Scientific (Illkirch, France)) and NGS assays (in house panel, sequencing platform from Illumina (San Diego, CA, USA), Process on GATK Joint Genotyping and MiSeqReporter version 2.4.6 software (Illumina, San Diego, CA, USA)) depending on the origin of the samples. A PCR-Restriction fragment length polymorphism assay was also used for *CYP3A5*.

#### 4.4.2. Comparison with Reference Methods Regarding Cost and Analytical Workflow

Because the objective of our assay was to be easy, fast, and cost-effective, we compared its properties regarding the analytical cost per patient, the workload for 10 samples and its general analytical performances, with those of NGS and Taqman^®^ technologies that are commonly used in laboratories. 

### 4.5. Clinical Cases

In order to illustrate the clinical interest of the present method, we reported the examples of three representative clinical cases. In these cases, pharmacogenetic analyses were asked by the physician to understand suboptimal drug response or onset of severe adverse events in patients. We reported our testimony of the analytical workflow before the implementation of the newly developed multiplex assay in our lab to emphasize the interest of the new method in regard to the clinical context. In particular, we underlined how the multiplex assay could have improved the time from prescription until results availability.

## 5. Conclusions

An accurate, rapid, and robust 17 variants-PCR multiplex genotyping assay was successfully developed. It includes a panel of 17 relevant variants to elucidate drug response variability. This assay can be easily implemented in laboratories as an alternative to simplex or expensive multiplex approaches. This Pharm-17-panel assay aims to widespread access to pharmacogenetics-based drug optimization in clinical practice.

## Figures and Tables

**Table 1:** Genetic variants of interest included in the panel of the multiplex assay.

Gene	rsID	Type of Polymorphism	Frequency of the Variant Allele *	Consequence of Allelic Variant on Protein Functionality **	Example of Drugs Potentially Impacted by the Polymorphism
*ABCB1*	rs1045642	SNP C > T	0.507	Reduced	Tacrolimus, Vincristine, Dabigatran, Some antipsychotics
*ABCB1*	rs1128503	SNP C > T	0.427	Reduced	
*ABCB1*	rs2032582	SNP G/T/A	T = 0.44 A = 0.001	Reduced	
*ABCB1*	rs2229109	SNP G > A	0.039	Reduced	
*CYP1A2*	rs762551 (*1F)	SNP C > A	0.681	Higher inducibility	Clozapine, Clopidogrel, Carbamazepine, Imatinib
*CYP2C9*	rs1799853 (*2)	SNP C > T	0.110	Reduced	Warfarin, phenytoin
*CYP2C9*	rs1057910 (*3)	SNP A > C	0.065	No function	
*CYP2C19*	rs4244285 (*2)	SNP G > A	0.150	No function	Clopidogrel, Citalopram, Escitalopram, Sertraline, Imipramine, protons pump inhibitors, Voriconazole
*CYP2C19*	rs4986893 (*3)	SNP G > A	0.008	No function	
*CYP2C19*	rs12248560 (*17)	SNP C > T	0.222	Increased	
*CYP2D6*	rs35742686 (*3)	delA	0.011	No function	Amitriptyline, Aripiprazole, Atomoxetine, Clomipramine, Codeine, Doxepin, Eliglustat, Flecainide, Haloperidol, Imipramine, Metoprolol, Nortriptyline, Paroxetine, Pimozide, Propafenone, Tamoxifen, Tramadol, Venlafaxine, Zuclopenthixol
*CYP2D6*	rs3892097 (*4)	C > T	0.182	No function	
*CYP2D6*	rs5030655 (*6)	delA	0.002	No function	
*CYP2D6*	rs5030656 (*9)	delAAG	0.019	Reduced	
*CYP3A4*	rs35599367 (*22)	C > T	0.042	Reduced	Tacrolimus, Statins
*CYP3A5*	rs776746 (*3)	A > G	0.887	No function	Tacrolimus
*VKORC1*	rs9923231	−1639 G > A	0.381	Reduced	Warfarin

* Frequencies according to dbSNP (ALFA) in global population, accessed on https://www.ncbi.nlm.nih.gov/snp/ (accessed on 9 July 2021). ** For CYP, clinical function of star alleles according to https://www.pharmvar.org. (accessed on 9 July 2021). CYP: CYP450, SNP: single nucleotide polymorphism, del: deletion. (*x): allele number.

**Table 2:** Influence of the amount of DNA analyzed on the performance of the analysis, expressed as peak intensity.

	DNA Amount						
Polymorphism	1 ng	2.5 ng	5 ng	10 ng	50 ng	250 ng	500 ng
*ABCB1-*rs1045642	59	97	69	437	1121	5151	5967
*ABCB1-*rs1128503	53	70	107	256	1119	2615	2638
*ABCB1*-rs2032582	89	134	110	440	1365	5003	4731
*ABCB1-*rs2229109	135	441	478	1241	4501	10,902	10,691
CYP1A2-*1F	56	164	131	649	1393	4320	3427
CYP2C9-*2	118	274	260	1174	2683	6604	5518
*CYP2C9*-*3	237	775	1011	2723	8621	15,517	14,752
CYP2C19-*2	52	144	137	554	1754	4465	4096
CYP2C19-*3	88	182	212	791	2134	4897	4023
CYP2C19-*17	63	228	222	748	2658	11,053	12,688
CYP2D6-*3	130	385	604	1266	5837	9932	11,488
CYP2D6-*4	N.D.	94	141	254	721	762	602
CYP2D6-*6	N.D.	100	242	304	2268	3437	4573
CYP2D6-*9	53	101	169	361	1385	2366	2675
*CYP3A4*-*22	143	356	352	1337	3553	8919	7619
CYP3A5-*3	189	534	543	1579	5141	11,242	10,717
*VKORC1-*rs9923231	136	315	386	948	3850	7534	7429

N.D.: undetermined; DNA: acid deoxyribonucleic. Results are expressed as peak intensity (height). A threshold of 100 intensity units is required to get a clear result. (*x): allele number.

**Table 3:** Stability of the ready-to-use pool of primers and probes.

Polymorphism	Mean Intensity with Fresh Pool	Mean Intensity with Pool FT5	Mean Bias (%) (FT5 vs. Fresh)
*ABCB1-*rs1045642	1205	1138	−6
*ABCB1-*rs1128503	1281	701	−45
*ABCB1-*rs2032582	2480	1793	−28
*ABCB1-*rs2229109	5049	4344	−14
CYP1A2-*1F	1660	1088	−34
CYP2C9-*2	3111	2120	−32
CYP2C9-*3	10,486	5711	−46
*CYP2C19*-*2	2073	1790	−14
CYP2C19-*3	2498	1100	−56
CYP2C19-*17	3004	2742	−9
CYP2D6-*3	7314	4187	−43
*CYP2D6*-*4	830	632	−24
CYP2D6-*6	3016	682	−77
*CYP2D6*-*9	1681	880	−48
CYP3A4-*22	4114	2841	−31
*CYP3A5*-*3	6126	4270	−30
*VKORC1-*rs9923231	4628	3015	−35

FT5: pool used after five freeze–thaw cycles. (*x): allele number.

**Table 4:** Accuracy of the assay, comparison of genotyping results with reference methods.

Gene	Genetic Polymorphism	Genotype	Number of DNA Samples (Multiplex Method)	Agreement with Reference Method
*CYP1A2*	rs762551 (C > A)	WT	15	100%
		varHz	21	100%
		varHm	13	100%
*CYP2C9*	rs1799853 (C > T)	WT	36	100%
		varHz	11	100%
		varHm	3	100%
	rs1057910 (C > T)	WT	36	100%
		varHz	12	100%
		varHm	1	100%
*CYP2C19*	rs4244285 (G > A)	WT	31	100%
		varHz	11	100%
		varHm	1	100%
	rs4986893 (G > A)	WT	31	100%
		varHz	1	100%
	rs12248560 (G > A)	WT	31	100%
		varHz	18	100%
		varHm	1	100%
*CYP2D6*	rs35742686 (A > G)	WT	22	100%
		varHz	3	100%
	rs3892097 (G > A)	WT	22	100%
		varHz	15	100%
		varHm	4	100%
	rs5030655 (A > C)	WT	22	100%
		varHz	4	100%
	rs5030656 (AAG > GAG)	WT	22	100%
		varHz	4	100%
*CYP3A4*	rs35599367 (C > T)	WT	101	100%
		varHz	14	100%
*CYP3A5*	rs776746 (T > C)	WT	3	100%
		varHz	37	100%
		varHm	81	100%
*VKORC1*	rs9923231 (G > A)	WT	9	100%
		varHz	10	100%
		varHm	9	100%
*ABCB1*	rs1045642 (A > G)	WT	17	100%
		varHz	39	100%
		varHm	33	100%
	rs1128503 (A > G)	WT	17	100%
		varHz	27	100%
		varHm	12	100%
	rs2032582 (T > G)	WT	13	100%
		varHz	23	100%
		varHm	18	100%
	rs2032582 (T > A)	WT	13	100%
		varHz	4	100%
	rs2229109 (C > T)	WT	48	100%
		varHz	9	100%

WT: wild-type allele; varHz: heterozygous for the variant allele; varHm: homozygous for the variant allele.

**Table 5:** Comparison of cost and analytical workflow with reference methods.

		TaqMan^®^ Genotyping Assays	Next Generation Sequencing	PCR Multiplex
***Cost* ^(1)^ *per patient for 17 variants ($)***	*For 1 sample*	199.5	1450	36.7
	*For 10 samples*	62.5	145	6
	*For 48 samples*	53.7	30	3
***Time to analyze 17 variants/samples (h)***	*Time of technical workload/10 samples*	5	4	1
	*Time in automate device/10 samples*	10	24	5
	*Time of analysis workload/10 samples*	2	1	0.5
	***Total***	**17**	**29**	**6.5**
***Analytical parameters***	*DNA sample amounts needed (ng)*	50	5	50
	*Stability of primer and fluorescent probes*	Good	Good	Good
	*Specificity*	Good	Excellent	Good
	*Sensitivity*	Good	Excellent	Good
	*Throughput*	Medium	High	Medium

^(1)^ These data are estimated based on our center experiences and usual literature reports. ($) are evaluated regarding cost of reagents and consumables. They do not include personal cost nor equipment acquisition since this part is inherent to laboratory policy. (h): hours.

## Data Availability

The datasets generated during and/or analyzed during the current study are available from the corresponding author on reasonable request.

## Supplementary Materials

The following are available online at https://www.mdpi.com/article/10.3390/ph15050637/s1, Figure S1: Principle of the assay, Table S1: Repeatability results, Table S2: Genotyping results of clinical cases and genes involved in the pharmacokinetics of treatments, Table S3. Design of primers used in the assay.
