# Supplementary material for: A Robust and Fast/Multiplex Pharmacogenetics Assay to Simultaneously Analyze 17 Clinically Relevant Genetic Polymorphisms in CYP3A4, CYP3A5, CYP1A2, CYP2C9, CYP2C19, CYP2D6, ABCB1, and VKORC1 Genes
Source: Pharmaceuticals (Basel). 2022 May 22;15(5):637. doi: 10.3390/ph15050637 (PMC9145594; doi:10.3390/ph15050637)
Supplement: Supplementary file 1 [file pharmaceuticals-15-00637-s001.zip › pharmaceuticals-1685628-supplementary.pdf]

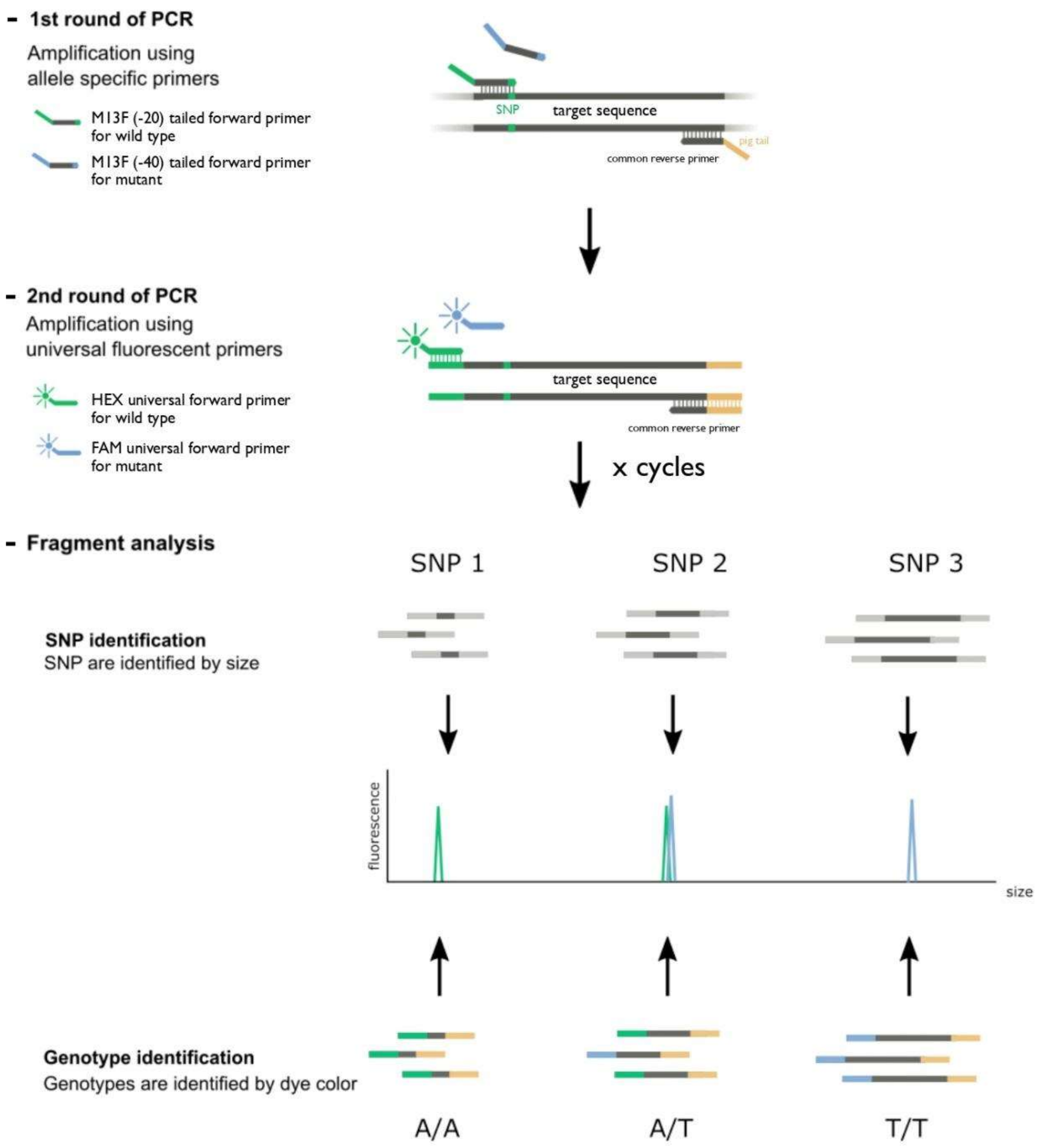

Figure S1: Principle of the assay

The first round of amplification used allele specific primers designed to obtain different fragment sizes for each genetic location. The last nucleotide of the 3'flanking forward primers was specific to the genotype (wild-type or a variant allele). The reverse primer was the same for both allele (wild-type or a variant allele). A Pigtail GTTTCTT was added to 5'flanking reverse primers to improve the amplicon migration. A second round of amplification used universal primers combined with fluorescent probes.

A M13 sequence (-20) GTAAAACGACGGCCAGT in the 5' flanking forward wild-type primers allowed hybridization and amplification of the wild type allele and a M13 sequence (-40) GTTTTCCCAGTCACGAC in the 5' flanking forward variant primers allowed hybridization and amplification of the variant allele. After denaturation, PCR products were loaded on a sequencer. The color of the fluorescence of the fragment allowed allelic discrimination and the size of the fragment allowed genetic position discrimination.

Table S1: Repeatability results. Eight internal control samples were analyzed in duplicate 4 different days.

QC1

	Day1		Day2		Day3		Day4		
position	status R1	status R2	status R1	status R2	status R1	status R2	status R1	status R2	repetability
ABCB1-rs1045642	HM	HM	HM	HM	HM	HM	HM	HM	100%
ABCB1-rs1128503	HM	HM	HM	HM	HM	HM	HM	HM	100%
ABCB1-rs2032582-A	DM	DM	DM	DM	DM	DM	DM	DM	100%
ABCB1-rs2032582-GT	DM	DM	DM	DM	DM	DM	DM	DM	100%
ABCB1-rs2229109	WT	WT	WT	WT	WT	WT	WT	WT	100%
CYP1A2-1F	DM	DM	DM	DM	DM	DM	DM	DM	100%
CYP2C9-2	HM	HM	HM	HM	HM	HM	HM	HM	100%
CYP2C9-3	WT	WT	WT	WT	WT	WT	WT	WT	100%
CYP2C19-2	WT	WT	WT	WT	WT	WT	WT	WT	100%
CYP2C19-3	WT	WT	WT	WT	WT	WT	WT	WT	100%
CYP2C19-17	HM	HM	HM	HM	HM	HM	HM	HM	100%
CYP2D6-3	WT	WT	WT	WT	WT	WT	WT	WT	100%
CYP2D6-4	HM	HM	HM	HM	HM	HM	HM	HM	100%
CYP2D6-6	WT	WT	WT	WT	WT	WT	WT	WT	100%
CYP2D6-9	WT	WT	WT	WT	WT	WT	WT	WT	100%
CYP3A4-22	WT	WT	WT	WT	WT	WT	WT	WT	100%
CYP3A5	DM	DM	DM	DM	DM	DM	DM	DM	100%
VKORC1-rs9923231	HM	HM	HM	HM	HM	HM	HM	HM	100%

QC2

	Day1		Day2		Day3		Day4		
position	status R1	status R2	status R1	status R2	status R1	status R2	status R1	status R2	repetability
ABCB1-rs1045642	HM	HM	HM	HM	HM	HM	HM	HM	100%
ABCB1-rs1128503	DM	DM	DM	DM	DM	DM	DM	DM	100%
ABCB1-rs2032582-A	-	-	-	-	-	-	-	-	100%

ABCB1-rs2032582-GT	DM	DM	DM	DM	DM	DM	DM	DM	100%
ABCB1-rs2229109	WT	WT	WT	WT	WT	WT	WT	WT	100%
CYP1A2-1F	HM	HM	HM	HM	HM	HM	HM	HM	100%
CYP2C9-2	WT	WT	WT	WT	WT	WT	WT	WT	100%
CYP2C9-3	WT	WT	WT	WT	WT	WT	WT	WT	100%
CYP2C19-2	WT	WT	WT	WT	WT	WT	WT	WT	100%
CYP2C19-3	WT	WT	WT	WT	WT	WT	WT	WT	100%
CYP2C19-17	DM	DM	DM	DM	DM	DM	DM	DM	100%
CYP2D6-3	WT	WT	WT	WT	WT	WT	WT	WT	100%
CYP2D6-4	DM	DM	DM	DM	DM	DM	DM	DM	100%
CYP2D6-6	HM	HM	HM	HM	HM	HM	HM	HM	100%
CYP2D6-9	WT	WT	WT	WT	WT	WT	WT	WT	100%
CYP3A4-22	WT	WT	WT	WT	WT	WT	WT	WT	100%
CYP3A5	DM	DM	DM	DM	DM	DM	DM	DM	100%
VKORC1-rs9923231	HM	HM	HM	HM	HM	HM	HM	HM	100%

QC3

	Day1		Day2		Day3		Day4		
position	status R1	status R2	status R1	status R2	status R1	status R2	status R1	status R2	repetability
ABCB1-rs1045642	DM	DM	DM	DM	DM	DM	DM	DM	100%
ABCB1-rs1128503	DM	DM	DM	DM	DM	DM	DM	DM	100%
ABCB1-rs2032582-A	-	-	-	-	-	-	-	-	100%
ABCB1-rs2032582-GT	DM	DM	DM	DM	DM	DM	DM	DM	100%
ABCB1-rs2229109	WT	WT	WT	WT	WT	WT	WT	WT	100%
CYP1A2-1F	DM	DM	DM	DM	DM	DM	DM	DM	100%
CYP2C9-2	DM	DM	DM	DM	DM	DM	DM	DM	100%
CYP2C9-3	WT	WT	WT	WT	WT	WT	WT	WT	100%
CYP2C19-2	WT	WT	WT	WT	WT	WT	WT	WT	100%
CYP2C19-3	WT	WT	WT	WT	WT	WT	WT	WT	100%
CYP2C19-17	WT	WT	WT	WT	WT	WT	WT	WT	100%
CYP2D6-3	WT	WT	WT	WT	WT	WT	WT	WT	100%
CYP2D6-4	WT	WT	WT	WT	WT	WT	WT	WT	100%
CYP2D6-6	WT	WT	WT	WT	WT	WT	WT	WT	100%
CYP2D6-9	WT	WT	WT	WT	WT	WT	WT	WT	100%
CYP3A4-22	HM	HM	HM	HM	HM	HM	HM	HM	100%
CYP3A5	DM	DM	DM	DM	DM	DM	DM	DM	100%
VKORC1-rs9923231	HM	HM	HM	HM	HM	HM	HM	HM	100%

QC4

	Day1		Day2		Day3		Day4		
position	status R1	status R2	status R1	status R2	status R1	status R2	status R1	status R2	repetability
ABCB1-rs1045642	HM	HM	HM	HM	HM	HM	HM	HM	100%
ABCB1-rs1128503	HM	HM	HM	HM	HM	HM	HM	HM	100%
ABCB1-rs2032582-A	-	-	-	-	-	-	-	-	100%
ABCB1-rs2032582-GT	HM	HM	HM	HM	HM	HM	HM	HM	100%
ABCB1-rs2229109	HM	HM	HM	HM	HM	HM	HM	HM	100%
CYP1A2-1F	DM	DM	DM	DM	DM	DM	DM	DM	100%
CYP2C9-2	WT	WT	WT	WT	WT	WT	WT	WT	100%
CYP2C9-3	HM	HM	HM	HM	HM	HM	HM	HM	100%
CYP2C19-2	WT	WT	WT	WT	WT	WT	WT	WT	100%
CYP2C19-3	WT	WT	WT	WT	WT	WT	WT	WT	100%
CYP2C19-17	WT	WT	WT	WT	WT	WT	WT	WT	100%
CYP2D6-3	WT	WT	WT	WT	WT	WT	WT	WT	100%
CYP2D6-4	HM	HM	HM	HM	HM	HM	HM	HM	100%
CYP2D6-6	WT	WT	WT	WT	WT	WT	WT	WT	100%
CYP2D6-9	HM	HM	HM	HM	HM	HM	HM	HM	100%
CYP3A4-22	WT	WT	WT	WT	WT	WT	WT	WT	100%
CYP3A5	DM	DM	DM	DM	DM	DM	DM	DM	100%
VKORC1-rs9923231	DM	DM	DM	DM	DM	DM	DM	DM	100%

QC5

	Day1		Day2		Day3		Day4		
position	status R1	status R2	status R1	status R2	status R1	status R2	status R1	status R2	repetability
ABCB1-rs1045642	HM	HM	HM	HM	HM	HM	HM	HM	100%
ABCB1-rs1128503	HM	HM	HM	HM	HM	HM	HM	HM	100%
ABCB1-rs2032582-A	-	-	-	-	-	-	-	-	100%
ABCB1-rs2032582-GT	HM	HM	HM	HM	HM	HM	HM	HM	100%
ABCB1-rs2229109	WT	WT	WT	WT	WT	WT	WT	WT	100%
CYP1A2-1F	WT	WT	WT	WT	WT	WT	WT	WT	100%
CYP2C9-2	WT	WT	WT	WT	WT	WT	WT	WT	100%
CYP2C9-3	WT	WT	WT	WT	WT	WT	WT	WT	100%
CYP2C19-2	WT	WT	WT	WT	WT	WT	WT	WT	100%
CYP2C19-3	WT	WT	WT	WT	WT	WT	WT	WT	100%
CYP2C19-17	HM	HM	HM	HM	HM	HM	HM	HM	100%
CYP2D6-3	WT	WT	WT	WT	WT	WT	WT	WT	100%
CYP2D6-4	WT	WT	WT	WT	WT	WT	WT	WT	100%
CYP2D6-6	WT	WT	WT	WT	WT	WT	WT	WT	100%
CYP2D6-9	WT	WT	WT	WT	WT	WT	WT	WT	100%
CYP3A4-22	WT	WT	WT	WT	WT	WT	WT	WT	100%
CYP3A5	WT	WT	WT	WT	WT	WT	WT	WT	100%
VKORC1-rs9923231	WT	WT	WT	WT	WT	WT	WT	WT	100%

QC6

	Day1		Day2		Day3		Day4		
position	status R1	status R2	status R1	status R2	status R1	status R2	status R1	status R2	repetability
ABCB1-rs1045642	HM	HM	HM	HM	HM	HM	HM	HM	100%
ABCB1-rs1128503	HM	HM	HM	HM	HM	HM	HM	HM	100%
ABCB1-rs2032582-A	-	-	-	-	-	-	-	-	100%
ABCB1-rs2032582-GT	HM	HM	HM	HM	HM	HM	HM	HM	100%
ABCB1-rs2229109	WT	WT	WT	WT	WT	WT	WT	WT	100%
CYP1A2-1F	HM	HM	HM	HM	HM	HM	HM	HM	100%
CYP2C9-2	WT	WT	WT	WT	WT	WT	WT	WT	100%
CYP2C9-3	WT	WT	WT	WT	WT	WT	WT	WT	100%
CYP2C19-2	HM	HM	HM	HM	HM	HM	HM	HM	100%
CYP2C19-3	WT	WT	WT	WT	WT	WT	WT	WT	100%
CYP2C19-17	WT	WT	WT	WT	WT	WT	WT	WT	100%
CYP2D6-3	HM	HM	HM	HM	HM	HM	HM	HM	100%
CYP2D6-4	WT	WT	WT	WT	WT	WT	WT	WT	100%
CYP2D6-6	WT	WT	WT	WT	WT	WT	WT	WT	100%
CYP2D6-9	WT	WT	WT	WT	WT	WT	WT	WT	100%
CYP3A4-22	WT	WT	WT	WT	WT	WT	WT	WT	100%
CYP3A5	DM	DM	DM	DM	DM	DM	DM	DM	100%
VKORC1-rs9923231	WT	WT	WT	WT	WT	WT	WT	WT	100%

QC7

	Day1		Day2		Day3		Day4		
position	status R1	status R2	status R1	status R2	status R1	status R2	status R1	status R2	repetability
ABCB1-rs1045642	DM	DM	DM	DM	DM	DM	DM	DM	100%
ABCB1-rs1128503	HM	HM	HM	HM	HM	HM	HM	HM	100%
ABCB1-rs2032582-A	-	-	-	-	-	-	-	-	100%
ABCB1-rs2032582-GT	HM	HM	HM	HM	HM	HM	HM	HM	100%
ABCB1-rs2229109	WT	WT	WT	WT	WT	WT	WT	WT	100%
CYP1A2-1F	HM	HM	HM	HM	HM	HM	HM	HM	100%
CYP2C9-2	WT	WT	WT	WT	WT	WT	WT	WT	100%
CYP2C9-3	WT	WT	WT	WT	WT	WT	WT	WT	100%
CYP2C19-2	DM	DM	DM	DM	DM	DM	DM	DM	100%
CYP2C19-3	WT	WT	WT	WT	WT	WT	WT	WT	100%
CYP2C19-17	WT	WT	WT	WT	WT	WT	WT	WT	100%
CYP2D6-3	WT	WT	WT	WT	WT	WT	WT	WT	100%
CYP2D6-4	HM	HM	HM	HM	HM	HM	HM	HM	100%
CYP2D6-6	WT	WT	WT	WT	WT	WT	WT	WT	100%
CYP2D6-9	WT	WT	WT	WT	WT	WT	WT	WT	100%

CYP3A4-22	WT	WT	WT	WT	WT	WT	WT	WT	100%
CYP3A5	HM	HM	HM	HM	HM	HM	HM	HM	100%
VKORC1-rs9923231	HM	HM	HM	HM	HM	HM	HM	HM	100%

QC8

	Day1		Day2		Day3		Day4		
position	status R1	status R2	status R1	status R2	status R1	status R2	status R1	status R2	repetability
ABCB1-rs1045642	WT	WT	WT	WT	WT	WT	WT	WT	100%
ABCB1-rs1128503	WT	WT	WT	WT	WT	WT	WT	WT	100%
ABCB1-rs2032582-A	-	-	-	-	-	-	-	-	100%
ABCB1-rs2032582-GT	WT	WT	WT	WT	WT	WT	WT	WT	100%
ABCB1-rs2229109	HM	HM	HM	HM	HM	HM	HM	HM	100%
CYP1A2-1F	HM	HM	HM	HM	HM	HM	HM	HM	100%
CYP2C9-2	WT	WT	WT	WT	WT	WT	WT	WT	100%
CYP2C9-3	WT	WT	WT	WT	WT	WT	WT	WT	100%
CYP2C19-2	WT	WT	WT	WT	WT	WT	WT	WT	100%
CYP2C19-3	HM	HM	HM	HM	HM	HM	HM	HM	100%
CYP2C19-17	HM	HM	HM	HM	HM	HM	HM	HM	100%
CYP2D6-3	WT	WT	WT	WT	WT	WT	WT	WT	100%
CYP2D6-4	WT	WT	WT	WT	WT	WT	WT	WT	100%
CYP2D6-6	WT	WT	WT	WT	WT	WT	WT	WT	100%
CYP2D6-9	WT	WT	WT	WT	WT	WT	WT	WT	100%
CYP3A4-22	HM	HM	HM	HM	HM	HM	HM	HM	100%
CYP3A5	DM	DM	DM	DM	DM	DM	DM	DM	100%
VKORC1-rs9923231	DM	DM	DM	DM	DM	DM	DM	DM	100%

WT: wild-type allele; varHz: heterozygous for the variant allele; varHm: homozygous for the variant allele
QC: internal quality control DNA
R: replicate

Table S2: Genotyping results of clinical cases and genes involved in the pharmacokinetics of treatments

	ABCB1-rs1045642	ABCB1-rs1128503	ABCB1-rs2032582-A	ABCB1-rs2032582-GT	ABCB1-rs2229109	CYP1A2*1F	CYP2C9*2	CYP2C9*3	CYP2C19*2	CYP2C19*3	CYP2C19*17	CYP2D6*3	CYP2D6*4	CYP2D6*6	CYP2D6*9	CYP3A4*22	CYP3A5*3	VKORC1-rs9923231
P1	WT	WT	**var Hm**	WT	WT	**var Hm**	WT	WT	WT	WT	**varHz**	**var Hz**	WT	WT	WT	WT	var Hm	**var Hz**
Haloperidol																		
Clozapine																		
P2	**var Hz**	**var Hz**	-	**var Hz**	WT	varHm	WT	WT	varHz	WT	WT	WT	WT	WT	WT	**var Hz**	var Hm	WT
P3	**var Hm**	**var Hm**	-	**var Hm**	WT	WT	var Hz	WT	WT	WT	WT	-	-	-	-	WT	var Hm	var Hz
Vincristine																		

Results of the PCR-Multiplex analysis

- Polymorphism on a gene not significantly involved in the drug pharmacokinetics
- Polymorphism on a gene moderately involved in the drug pharmacokinetics
- Polymorphism on a gene strongly involved in the drug pharmacokinetics

P1, P2, P3: patient ID

WT: wild-type allele; varHz: heterozygous for the variant allele; varHm: homozygous for the variant allele

(-): no amplification because of full gene deletion

Table S3. Design of primers used in the assay

Gene and variant	Primer ID	Primer sequences (5'-3')	amplicon size (pb)	Primer final concentration in the mix (pmol/µL)
CYP1A2*1F rs762551	F-CYP1A2*1F -WT	**GTAAAACGACGGCCAGT**CAAAGGGTGAGCTCTGTGGTCC	160	0.02
	F-CYP1A2*1F -var	**GTTTTCCCAGTCACGAC**CAAAGGGTGAGCTCTGTGGACA		0.02
	R-CYP1A2*1F	**GTTTCTT**TGGAGACATTCATTCATTTCC		0.3
CYP2C9*2 rs1799853	F-CYP2C9*2-WT	**GTAAAACGACGGCCAGT**GGGAAGAGGAGCATTGAGCACC	127	0.02
	F-CYP2C9*2-var	**GTTTTCCCAGTCACGAC**GGGAAGAGGAGCATTGAGCACT		0.01
	R-CYP2C9*2	**GTTTCTT**AGGTCAGTGATATGGAGTAGGGT		0.2
CYP2C9*3 rs1057910	F-CYP2C9*3-WT	**GTAAAACGACGGCCAGT**GTGCACGAGGTCCAGAGAAACA	328	0.03
	F-CYP2C9*3-var	**GTTTTCCCAGTCACGAC**GTGCACGAGGTCCAGAGAAACC		0.01
	R-CYP2C9*3	**GTTTCTT**TAAATCTGGAGAACACACACTGCC		0.4
CYP2C19*2 rs4244285	F-CYP2C19*2-WT	**GTAAAACGACGGCCAGT**TTCCCACTATCATTGATTATTTGCCG	164	0.01
	F-CYP2C19*2-var	**GTTTTCCCAGTCACGAC**TTCCCACTATCATTGATTATTTGCCA		0.01
	R-CYP2C19*2	**GTTTCTT**ATCAATAAAGTCCCGAGGGTTGT		0.4
CYP2C19*3 rs4986893	F-CYP2C19*3-WT	**GTAAAACGACGGCCAGT**GGATTGTAAGCACCCCGTGG	183	0.01
	F-CYP2C19*3-var	**GTTTTCCCAGTCACGAC**GGATTGTAAGCACCCCGTGA		0.005
	R-CYP2C19*3	**GTTTCTT**TCACCCCATGGCTGTCTAGG		0.2
CYP2C19*17 rs12248560	F-CYP2C19*17-WT	**GTAAAACGACGGCCAGT**GGCGCATTATCTCTTACATCAGACATG	202	0.04
	F-CYP2C19*17-var	**GTTTTCCCAGTCACGAC**GGCGCATTATCTCTTACATCAGACATA		0.01
	R-CYP2C19*17	**GTTTCTT**GTGGTTCTATTTAATGTGAAGCCTGTTTTATG		0.8
CYP2D6*3 rs35742686	F-CYP2D6*3-WT	**GTAAAACGACGGCCAGT**GATGAGCTGCTAACTGAGGACA	478	0.02
	F-CYP2D6*3-var	**GTTTTCCCAGTCACGAC**GATGAGCTGCTAACTGAGGACG		0.005
	R-CYP2D6*3	**GTTTCTT**CGGCCCCTGCACTGTTTC		0.2
CYP2D6*4 rs3892097	F-CYP2D6*4-WT	**GTAAAACGACGGCCAGT**CCGCATCTCCCACCCTCAG	450	0.02
	F-CYP2D6*4-var	**GTTTTCCCAGTCACGAC**CCGCATCTCCCACCCTCAA		0.01
	R-CYP2D6*4	**GTTTCTT**TCTCTGACGTGGATAGGAGGTACA		1.6
CYP2D6*6 rs5030655	F-CYP2D6*6-WT	**GTAAAACGACGGCCAGT**CCTCCTCGGTCAGCCCA	227	0.04
	F-CYP2D6*6-var	**GTTTTCCCAGTCACGAC**CCTCCTCGGTCAGCCCC		0.01
	R-CYP2D6*6	**GTTTCTT**CCAGGGGGAGCATAGGGTT		2
CYP2D6*9 rs5030656	F-CYP2D6*9-WT	**GTAAAACGACGGCCAGT**CTTCCTGGCAGAGATGGACAAG	410	0.02
	F-CYP2D6*9-var	**GTTTTCCCAGTCACGAC**CCTTCCTGGCAGAGATCGAG		0.01
	R-CYP2D6*9	**GTTTCTT**CGGCCCCTGCACTGTTTC		1.6

CYP3A4*22 rs35599367	F-CYP3A4*22-WT	**GTAAAACGACGGCCAGT**AGTGTCT CCATCACACC**G**AGC	118	0.02
	F-CYP3A4*22-var	**GTTTTCCCAGTCACGAC**AGTGTCTC CATCACACC**G**AGT		0.01
	R-CYP3A4*22	**GTTTCTT**GATCTACTAGATCACCTTC TATCACACTCCA		0.4
CYP3A5*3 rs776746	F-CYP3A5-WT	**GTAAAACGACGGCCAGT**TGTGGTC CAAACAGGGAAGAG**T**TAT	305	0.02
	F-CYP3A5-var	**GTTTTCCCAGTCACGAC**TGTGGTCC AAACAGGGAAGAG**T**TAC		0.01
	R-CYP3A5	**GTTTCTT**AGATGACACAGCTCTAGA TGTCC		0.8
VKORC1-rs9923231	F-VKORC1-rs9923231-WT	**GTAAAACGACGGCCAGT**GACCTGA AAAACAACCATTG**G**ACG	397	0.01
	F-VKORC1-rs9923231-var	**GTTTTCCCAGTCACGAC**GACCTGAA AAACAACCATTG**G**ACA		0.04
	R-VKORC1-rs9923231	**GTTTCTT**CCTGACACCTAGTGGCTG GT		1.6
ABCB1-rs1045642	F-ABCB1-rs1045642-var	**GTAAAACGACGGCCAGT**CTCCTTTG CTGCCCT**G**ACA	319	0.02
	F-ABCB1-rs1045642-WT	**GTTTTCCCAGTCACGAC**CTCCTTTG CTGCCCT**G**ACG		0.005
	R-ABCB1-rs1045642	**GTTTCTTT**CACACAAACTTTTCCTTA ATCTCA		0.8
ABCB1-rs1128503	F-ABCB1-rs1128503-var	**GTAAAACGACGGCCAGT**ACTCTGC ACCTTCAGGTT**G**AGA	338	0.02
	F-ABCB1-rs1128503-WT	**GTTTTCCCAGTCACGAC**ACTCTGCA CCTTCAGGTT**G**AGG		0.005
	R-ABCB1-rs1128503	**GTTTCTT**AGCCAAGTATTGACAGCT ATTCG		0.6
ABCB1-rs2032582	F-ABCB1-rs2032582-var-T	**GTAAAACGACGGCCAGT**ATTTAGTT TGACTCACCTTCC**G**AGA	424	0.02
	F-ABCB1-rs2032582-WT-G	GTTTTCCCAGTCACGACATTTAGTTT GACTCACCTTCC**G**AGC		0.005
	F-ABCB1-rs2032582-var-A	**GTTTTCCCAGTCACGAC**CATATTTA GTTTGACTCACCTTCCC**T**GT	427	0.005
	R-ABCB1-rs2032582	**GTTTCTT**CTGAAGTCATGGAAATTCT TACTGT	424/427	0.8
ABCB1-rs2229109	F-ABCB1-rs2229109-WT	**GTAAAACGACGGCCAGT**CCTTAACT TCTTTTCGAGATGGG**A**AAC	360	0.02
	F-ABCB1-rs2229109-var	**GTTTTCCCAGTCACGAC**CCTTAACT TCTTTTCGAGATGGG**A**AAT		0.01
	R-ABCB1-rs2229109	**GTTTCTT**GGACAGGCATCTCCAAGC AT		0.4

F: forward; R: reverse; WT: wild type; var: variant
GTAAAACGACGGCCAGT: M13 sequence (-20) added to 5' flanking forward primers
GTTTTCCCAGTCACGAC: M13 sequence (-40) added to the 5' flanking forward primers
GTTTCTT: "Pigtail" added to 5'flanking reverse primers
X: mismatch nucleotide in bold
X: position of the polymorphism
For the design of primers, the term "variant" was assigned to the lowest frequent allele compared to wild type in the total human population and according to the database dbSNP https://www.ncbi.nlm.nih.gov/snp/
